# Surface Analysis of Ti-Alloy Micro-Grooved 12/14 Tapers Assembled to Non-Sleeved and Sleeved Ceramic Heads: A Comparative Study of Retrieved Hip Prostheses

**DOI:** 10.3390/ma16031067

**Published:** 2023-01-25

**Authors:** Andrea Martelli, Paolo Erani, Nicola Pazzagli, Valeria Cannillo, Massimiliano Baleani

**Affiliations:** 1 Laboratorio di Tecnologia Medica, IRCCS Istituto Ortopedico Rizzoli, 40136 Bologna, Italy; 2 Dipartimento di Ingegneria Enzo Ferrari, Università degli Studi di Modena e Reggio Emilia, 41125 Modena, Italy

**Keywords:** 12/14 taper, adapter sleeve, ceramic heads, fretting corrosion, retrieval analysis, sleeved ceramic heads, titanium alloy, total hip arthroplasty

## Abstract

Ti6Al4V titanium alloy (Ti-alloy) sleeved ceramic heads have become widely used in revision surgery when the hip stem is left in situ. This solution guarantees a new junction between the bore of the ceramic head and the Ti-alloy sleeve, regardless of any possible, slight surface damage to the Ti-alloy taper of the stem. However, this solution introduces an additional Ti-alloy/Ti-alloy interface pairing, which is potentially susceptible to mechanically assisted crevice corrosion. This study evaluated both qualitatively and quantitatively the damage that occurred in vivo on Ti-alloy micro-grooved 12/14 tapers of (i) primary implants with non-sleeved ceramic heads (Group 1), (ii) secondary implants with non-sleeved ceramic heads (Group 2), and (iii) secondary implants with sleeved ceramic heads (Group 3). A total of 45 explants—15 for each group, including short-, medium- and long-neck heads—underwent optical evaluation for surface damage (Goldberg scoring), surface roughness analysis, and SEM/EDX analysis. The Goldberg scores did not reveal different patterns in the tapers’ surface damage; surface damage was classified as absent or mild (surface damage score ≤2) in 94%, another 94%, and 92% of the analysed regions for Group 1, Group 2, and Group 3, respectively. Small but significant differences in morphological changes occurred in the tapers of the three groups: reductions no greater than a few percentage points in median values of roughness parameters were found in Group 1 and Group 2, while negligible changes were found in Group 3. SEM/EDX analysis revealed little (i.e., a slight increase in the oxygen content) to undetectable changes in the chemical composition on the Ti-alloy surface independently of the group. These results suggest that the Ti-alloy/Ti-alloy sleeve/taper junction is only mildly susceptible to mechanically assisted crevice corrosion. Assembling a sleeved ceramic head, with variable neck lengths up to a “long-neck”, to a Ti-alloy micro-grooved 12/14 taper of a stem left in situ does not seem to increase the risk of revision due to trunnionosis, as long as junction stability (i.e., the proper seating of the sleeved ceramic head on the 12/14 taper) is achieved intraoperatively.

## 1. Introduction

Modern hip replacement designs commonly include one or more modular components. Modular designs allow for the manufacture of each prosthetic component using the most suitable material for the given in vivo function and, to a certain extent, the adaptation of the hip prosthesis to the patient’s anatomy.

Among the available solutions, a surgeon can opt for a non-sleeved ceramic head to be attached to the femoral component, which is commonly made of Ti6Al4V titanium alloy (hereinafter referred to as Ti-alloy) [1,2]. Attachment is achieved intraoperatively by fitting the head onto a taper. At time of writing, the 12/14 taper is the most commonly used design in Europe, although other designs are available [3].

Retrieval analysis and clinical outcomes show that the non-sleeved ceramic head/Ti-alloy taper junction is less prone to in vivo damage compared to other material combinations. Koganoz et al. [4] analysed a matched cohort of 50 ceramic and 50 metal head–stem pairs. They found that the cumulative volumetric material loss from the ceramic taper junctions was significantly less than in the CoCr taper junctions. Baleani et al. [5] analysed 148 retrieved implants, of which 61 included a ceramic head and 87 a metal head. They showed that chemical phenomena within the head/taper junction were mitigated by the use of a ceramic femoral head. In an analysis of data from published studies, Berstock et al. [6] concluded that “*the use of ceramic compared with metal heads in the primary setting significantly reduces the incidence of trunnionosis*”, i.e., a complication of hip arthroplasty caused by products of in vivo damage of the head/neck junction. More recently, Eichler et al. [7] reported no radiological or biological signs of trunnionosis with large-diameter ceramic heads and Ti-alloy femoral stems.

Several key factors affect junctional stability and, ultimately, the risk of in vivo junction damage and consequent trunnionosis. The most important of these is taper mismatch due to manufacturing tolerances [8,9]. Manufacturing tolerances play a crucial role in determining micromotion amplitude at the head/taper interface [10,11]. Taper mismatch also has a significant effect on the risk of the fracture of non-sleeved ceramic heads, leading to higher stress in localised areas, i.e., stress concentration [12]. A micro-grooved taper finish has been proposed to minimise the effect of manufacturing tolerances on the head/taper junction behaviour. Plastic deformation of the ridges at the time of the head’s assembly limits the concentration of stresses on the ceramic head and guarantees junctional stability [13,14]. This mechanism may be less effective in the case of head revision. Indeed, the ridge plasticization process is irreversible. Therefore, the attachment of a new head to a used taper could lead to suboptimal seating conditions at the head–neck interface, with undesirable stress concentrations in the ceramic material [15,16].

In the last decade, Ti-alloy adapter sleeves have been widely used as components with which to line the bores of revision ceramic heads (sleeved ceramic head) [17]. This solution guarantees a new junction and, therefore, pristine conditions between the head bore and the outer surface of the sleeve, regardless of possible damage to the taper of the prosthetic stem left in situ. On the other hand, this solution introduces an additional Ti-alloy/Ti-alloy junction between the sleeve and the taper of the stem. When the micromovement amplitude is in the order of magnitude of tens of microns [18,19,20,21,22], the Ti-alloy/Ti-alloy junction provides good resistance to mechanically assisted crevice corrosion (MACC). However, there is a risk that microinstability, i.e., interface micromovement, at the sleeve/taper interface could be greater when the sleeve is coupled with a used taper (even when apparently in pristine conditions) compared to that found at a sleeve/new-taper interface. The magnitude of the micromovement within the junction is one of the key factors affecting MACC, along with the implantation time, the stress level within the Ti-alloy, and interface friction conditions [23,24,25,26,27]. The occurrence of MACC may promote the release of metal debris in the periprosthetic tissues or ion release at the systemic level [5,28,29], thus increasing the risk of implant failure [3,30,31,32].

To our knowledge, only three studies have evaluated the damage caused in vivo at the sleeve/taper junction. MacDonald et al. [33] analysed 37 retrieved sleeved ceramic heads with an average implantation time of 0.7 years (range: 0.0–3.3 years). Their observations were limited to a qualitative evaluation using the grading system proposed by Goldberg at al. [34]. They reported Goldberg scores for internal tapers and femoral stems (only available for 18 out of 37 cases) higher than those for the external sleeve taper. Koch et al. [35] analysed 24 retrieved sleeved ceramic heads and femoral stems (only available for 7 out of 24 cases) with an average implantation time of 1.3 years (range: 0.1–5.4 years). The taper analysis included Goldberg scoring, scanning electron microscopy (SEM), and energy dispersive X-ray (EDX) analysis. They confirmed the observations of MacDonald et al., while SEM and EDX analyses provided further indications of low levels of damage. However, both studies analysed retrievals with a short implantation time and did not include a control group. Wyles et al. [36] carried out a retrieval study including both qualitative and quantitative evaluations. Ten primary implants, with an average implantation time of 4.0 years (range: 0.4–11.3 years), and six revised implants, with an average implantation time of 8.0 years (range: 0.4–20.1 years), were analysed. The comparison between the two groups suggested that the sleeves assembled with the used tapers may undergo greater in vivo damage than those assembled with new tapers, although no significant difference was found regarding surface damage. However, due to the limited sample size of the retrieved implants (three out six revisions involved the revision of the stem leading to a new sleeve/taper interface), a robust statistical analysis was precluded, as acknowledged by the same authors. Therefore, few data are available regarding the taper of the stem—the only component of the sleeve/taper junction that may have preexisting damage when left in situ at the time of revision.

We hypothesised that any light damage on the taper surface of the stem left in situ could increase the risk of in vivo damage of the sleeve/taper junction. Retrieved secondary implants with a sleeved ceramic head, which all included the stem, were paired with primary and secondary implants without the adapter sleeve, which all included the stem, to achieve a matched cohort. In vivo damage that occurred on the stem tapers was compared to assess the differences (if any) in the damage patterns among the three groups.

## 2. Materials and Methods

### 2.1. Retrieved Implants

All retrieved implants analysed in the present study were collected within the frame of the Register of Explanted Orthopaedic Prostheses (REPO) project. The project requires that all hip prostheses retrieved in our institute are registered and available for retrieval studies. The inclusion criteria used to select the secondary implants with sleeve/taper junction were as follows:

1. Head must be made of fourth-generation ceramic;
2. Head must have a sleeve made of Ti-alloy with a 12/14 internal taper (sleeved ceramic head);
3. Stem must have a micro-grooved 12/14 taper and be made of Ti-alloy.

The last criterion was included to avoid bias due to flexural rigidity of different taper designs. A total of 15 retrieved implants were eligible for this study. All implants were cementless. The primary and secondary implants without an adapter sleeve (non-sleeved ceramic head) were selected using the following criteria:

1. Head must be made of fourth-generation ceramic;
2. Stem must have a micro-grooved 12/14 taper and be made of Ti-alloy;
3. The implant must be cementless.

A total of 256 retrieved implants fulfilled the three aforementioned criteria. In order to select 15 primary and secondary implants, i.e., the same sample size of the implants including sleeved ceramic heads, three additional selection criteria were used:

4. The total implantation time must be comparable, i.e., with a difference smaller than one year, to that of the corresponding implant with sleeved ceramic head;
5. Patient body mass index (BMI) must be as close as possible to that of the patient from whom the corresponding implant with the sleeve/taper junction was retrieved;
6. Patient age must be as close as possible to that of the patient from whom the corresponding implant with the sleeve/taper junction was retrieved.

These three additional criteria were defined assuming that comparable follow-up, patient BMI and patient age would determine similar in vivo load history for the retrieved implants of the three groups (Group 1—primary implants including a non-sleeved ceramic head; Group 2—secondary implants including a non-sleeved ceramic head; Group 3—secondary implants including a sleeved ceramic head). The workflow is illustrated in Figure S1.

Clinical information and ceramic head configurations are listed in Table 1. All retrieved implants were collected and cleaned such that any biological fluids or tissue residuals were removed. Twelve heads were still attached to their stems and were disassembled in the laboratory.

### 2.2. Surface Damage Scoring

Any damage that occurred in vivo in the head/taper or sleeve/taper junctions was evaluated using the four-point scoring system based on damage morphology and extension proposed by Goldberg and co-workers [34]. All 12/14 taper surfaces underwent optical microscopy inspection. Taper surface was visually inspected under a stereomicroscope (SMZ-2T stereomicroscope, Nikon, Tokyo, Japan). The inspection was performed at up to 50X magnification when a detailed inspection was required. Three operators (PE, NP, and MB) independently evaluated the 12/14 taper surface under blind conditions. The surface inspection was carried out on the medial, anterior, lateral, and posterior quadrants, which were further divided into three equal regions along the taper axis, referred to as proximal, middle, and distal regions. Therefore, 12 inspections were performed on each taper surface. When the assessment differed among the three operators, a consensus was reached through open discussion while performing simultaneous inspections of the taper surface.

### 2.3. Surface Roughness Measurement

The 12 regions of each previously inspected 12/14 taper underwent roughness analysis. Surface roughness was measured along three circumferentially spaced generatrixes, i.e., along the axis direction of the taper, avoiding scratches (if any) generated during implant retrieval. The average value of the three measurements was used in statistical analysis. Roughness measurements were performed using a mechanical tester (Hommel Tester T8000, Hommelwerke-Jenoptik, Jena, Germany) equipped with a stylus having a tip radius of 2 µm and a tip angle of 90°. The travelling speed of the stylus was 0.05 mm/s. The evaluation length was set to 5 cut-offs (sampling lengths), with a cut-off length of 0.8 mm and a cut-off ratio of 100:1. The cut-off filter was the ISO Gaussian type. The acquisition range was 80 µm, determining a resolution of 0.01 µm. When the engagement length of the 12/14 taper was three times shorter than the travelling length, i.e., 18 cut-offs, the start or the end of the travelling length were overlapped—by up to 1.2 mm in tapers with the shortest engagement length. For each profile, the arithmetic average of the absolute values of the profile heights (Ra), the root-mean-square average of the profile heights (Rq), and the average maximum height of the profile (Rz) were calculated. The same roughness analysis was carried out on new 12/14 tapers to determine the percentage changes in roughness parameters due to in vivo damage of the taper surface, and, therefore, eliminate any potential bias due to small differences in original surface texture. Calibration checks were performed before each session using two certified reference profiles (Ra = 0.5 µm and Ra = 3.2 µm) to ensure that there were no drifts in the measuring equipment.

### 2.4. Scanning Electron Microscope Analysis

Three implants for each group were selected among those showing higher damage levels for SEM observations (Zeiss EVO MA10, Carl Zeiss, Oberkochen, Germany) and EDX analysis (Oxford INCA energy 200, Oxford Instruments Analytical, Wycombe, UK). The tapers were cut from the stems and cleaned to render the samples suitable for SEM analysis. The cleaning procedure involved placing each taper in detergent solution and then washing it in an ultrasonic bath for 10 min at 70 °C. The tapers were then placed in distilled water and washed again for 10 min at 70 °C. SEM and EDX analyses were carried out to assess changes in Ti-alloy surface morphology and composition due to in vivo damage.

### 2.5. Statistical Analysis

Log-linear analysis was used to examine differences between Goldberg scores. The Kruskal–Wallis test, followed by an adjusted pairwise comparison (APC), was used to investigate differences in roughness parameters. Both statistical tests were performed using a free software environment [37]. The significant level (*p*-value) for all statistical analyses was set at 0.05.

## 3. Results

### 3.1. Surface Damage Scoring

**Figure 1:**
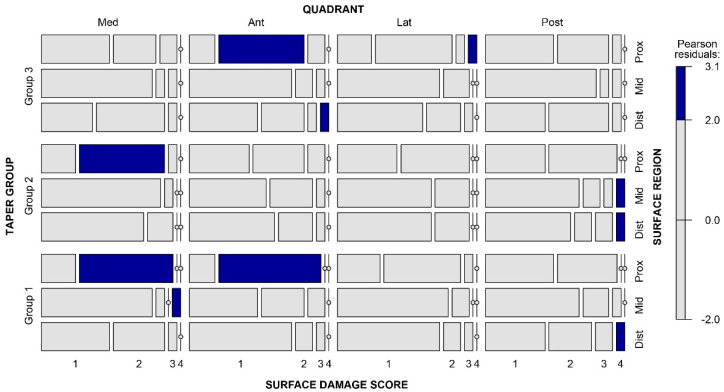
Mosaic plot showing the frequency of surface damage scores for the 12 regions of the taper surface. The tile width represents the number of the score value in each cell. The colour of each tile corresponds to the value of Pearson residuals. The bar on the right shows the correspondence between colours and values of Pearson residuals and the cut-off points.

The surface damage of the retrieved tapers was generally low. Surface damage was classified as absent or mild (surface damage score ≤2) in 94%, 94%, and 92% of the analysed regions for Group 1, Group 2, and Group 3, respectively. Severe damage (a surface damage score of 4) was found in six cases, with two in each group. In such cases, localised flattened areas with nearby fretting scars, without signs of chemical damage, were observed. Seemingly, the proximal regions of the tapers were more likely to be damaged (Figure S3). However, the level of Pearson residuals appeared quite homogeneous across the different regions despite the cell (Figure 1), i.e., no statistically significant differences were found within the distribution of the surface damage score values (*p* = 0.08).

### 3.2. Surface Roughness Measurement

A total of 1587 surface roughness measurements out of 1620 nominal ones were performed. Surface roughness was not measured in 11 out of 540 regions due to the local presence of one or more deep grooves running through several ridges, generally sloped towards the taper axis. None of the stems disassembled in the laboratory showed such surface scratches.

**Figure 2:**
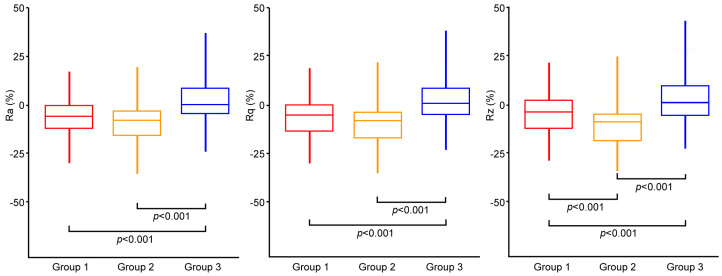
Box plot showing the distribution of the percentage change for Ra, Rq, and Rz parameters among the three groups. *p*-values of APC are reported.

The percentage changes in the roughness parameters due to the in vivo damage of the taper surface are shown in Figure 2. A small reduction was found in Groups 1 and 2 despite the measured parameters. The changes found in Group 3 were smaller, or even negligible, compared to those found in Groups 1 and 2. Indeed, a significant difference was found in the percentage changes among the groups (Kruskal–Wallis: *p* < 0.001 in all cases). The percentage changes in Group 2 were significantly lower than those measured in Group 3 independently of the investigated parameter (APC: *p* < 0.001 in all cases). The same consideration applies to the comparison between Groups 1 and 3 (APC: *p* < 0.001 in all cases). Significant differences between Groups 1 and 2 were found to be limited to the Rz parameter (APC: *p* < 0.001). Three representative roughness profiles are shown in Figure S4.

### 3.3. Scanning Electron Microscope Analysis

**Figure 3:**
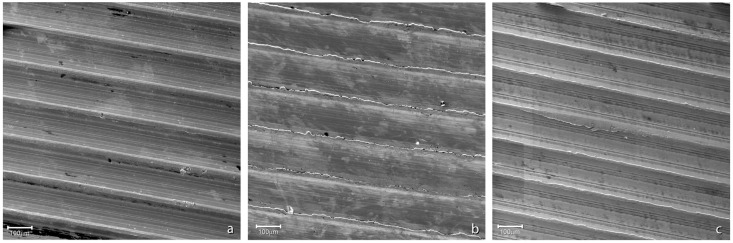
SEM images of tapers’ surfaces (magnification: 250×, working distance: 14 mm; accelerating voltage: 20.00 kV): (**a**) taper of Group 1; (**b**) taper of Group 2; (**c**) taper of Group 3.

**Figure 4:**
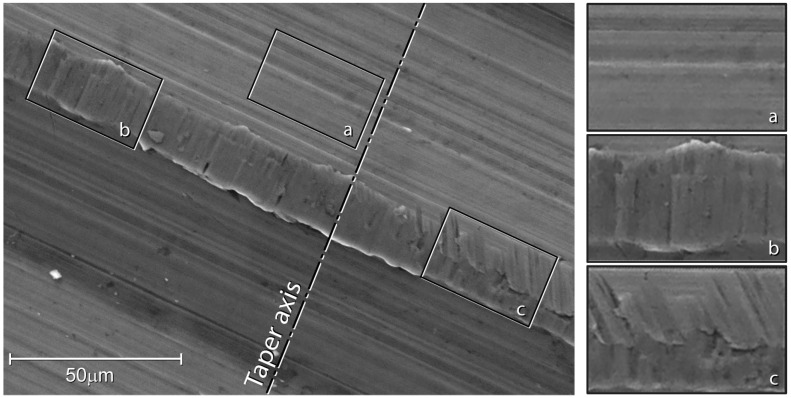
SEM image of taper surface (magnification: 1600×, working distance: 10.7 mm; accelerating voltage: 20.00 kV). Detail (**a**): original surface left from the cutting tool. Detail (**b**): parallel scars aligned with the taper axis, likely caused by fretting. Detail (**c**): parallel scar sloped towards the taper axis, which was likely generated during head disassembly.

**Figure 5:**
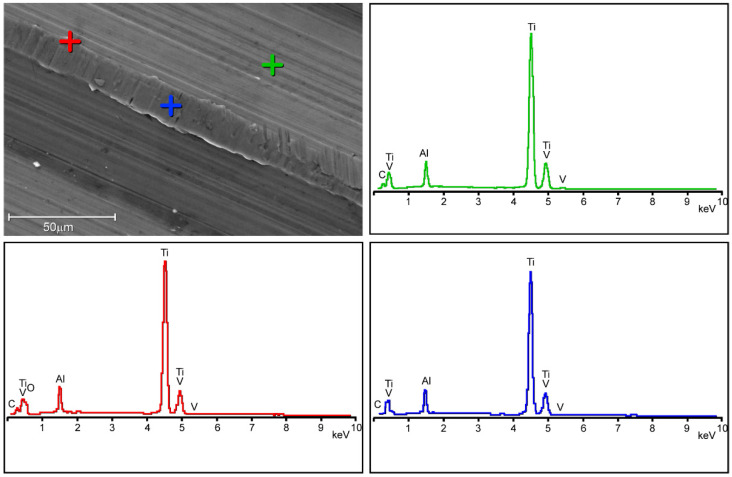
Same SEM image as the previous figure. EDX spectra acquired between the ridges (green spectrum), in the middle of the ridge (blue spectrum), and at the edge of the ridge (red spectrum).

Surface damage was not homogeneously distributed over the 12/14 taper surfaces. In general, surface damage was located on the ridges (Figure 3). The ridges often appeared plasticised. Finely spaced parallel micro-marks, aligned with the taper axis, were often found on the tip of the plasticised region (Figure 4). Little to no damage was observed between ridges where parallel micro-grooves, running aligned with ridges left on the surface by the cutting tool, were generally visible (Figure 4). Parallel scars sloped to the taper axis, likely generated during head disassembly, were also found in some areas. The above-mentioned micro-grooves aligned with the taper axis were no longer visible in these regions (Figure 4). No evidence of corrosion was observed at magnification levels up to 1600×. EDX spectra acquired between the ridges showed peaks corresponding to the Ti-alloy elements, i.e., Ti, Al, and V (Figure 5). Similar spectra were acquired on the tips of the plasticised areas, where micro-grooves aligned with the taper axis were visible. Some traces of oxygen were detected on the edges of the plasticised regions.

## 4. Discussion

This study investigated the damage that occurred in vivo on Ti-alloy micro-grooved 12/14 tapers. The in vivo damage in secondary implants with sleeved ceramic heads was compared with that found in primary and secondary implants with non-sleeved ceramic heads. Qualitative and quantitative evaluations were carried out on 45 retrievals to test the hypothesis that the light damage on the taper surface of the stem left in situ could increase the risk of in vivo damage of the sleeve/taper junction.

The optical evaluation of surface damage using the score proposed by Goldberg et al. [34] did not reveal specific patterns in the surface damage of the 12/14 taper. In general, the damage was classified from mild to absent (Goldberg score ≤2), independently from the group. Four previous studies provide confirmation of these observations. Kurtz et al. [38] have analysed, among the investigated material combinations, non-sleeved ceramic heads attached to Ti-alloy tapers with an average implantation time of 3.8 years (range: 0.6–17.8 years). The authors have assigned in all cases, except one, Goldberg scores between 1 and 2. Similar findings were reported in another study investigating retrieved sleeved ceramic heads attached to Ti-alloy tapers. Indeed, MacDonald et al. [33] have reported Goldberg scores ≤ 2 in 94% of the analysed Ti-alloy tapers with an average implantation time of 0.7 years (range: 0.0–3.3 years). Koch et al. [35], investigating the same configuration (sleeved ceramic head/Ti-alloy taper) with an average implantation time of 1.7 years (range: 0.1–5.5 years), have also reported a mean Goldberg score of 1.8 for the five analysed Ti-alloy tapers. Wyles et al. [36] have reported Goldberg scores between 1 and 2 for 10 out of 15 Ti-alloy tapers assembled with sleeved ceramic heads (average implantation time of 5.2 years; range: 0.4–20.1 years). However, a Goldberg score of 3 has been assigned to the remaining five retrieved tapers. The overall picture seems to suggest that the insertion of a Ti-alloy sleeve into the ceramic head bore has little effect on the risk of the occurrence of MACC within the Ti-alloy/Ti-alloy sleeve/taper junction. However, taper roughness seems to play a role in determining the extent of taper damage. Indeed, Stockhausen et al. [39], analysing ceramic heads directly attached to Ti-alloy tapers for an average implantation time of 7.3 years, found significant differences between deeply and lightly threaded tapers, with lower Goldberg scores for lightly threaded tapers coupled with ceramic heads. Notably, the micro-grooved tapers investigated in the present study were comparable to lightly threaded tapers in terms of their surface roughness.

The visual scoring method is not quantitative and cannot capture subtle changes that occur in taper surface morphology. To our knowledge, only two studies measured the loss of taper material due to damage that occurred in vivo in implants with ceramic heads. In the aforementioned study of Stockhausen et al. [39], a reduction up to about 5 µm in the thread heights of lightly threaded tapers was estimated. On the other hand, Wyles et al. [36] reported that *“approximately 1/3 of the retrievals developed maximum linear material loss >10 microns*”. However, graphical representations suggest that both positive and negative variations in profile heights may occur when the Ti-taper is assembled through a Ti-alloy sleeve to the ceramic head. All these findings are in agreement with the roughness changes found in this study. Indeed, we found a reduction in the roughness parameters in Groups 1 and 2, while the variation in Group 3 fluctuated around zero percent. This proves that both positive and negative variations in profile heights occurred. It seems likely that the abrasive wear of the Ti-alloy surface due to micromotions occurring at the ceramic/Ti-alloy interface [40], which leads to metal removal and the one-way transfer of material (i.e., metal transfer to the ceramic surface) [5,38,41], might switch toward more mutual damage when both surfaces are made of Ti-alloy [36].

Whichever one was the damage mechanism, taper damage was limited to the surface peaks. Indeed, SEM observations revealed that alterations in taper surface morphology were generally limited to ridges. This pattern was consistent across all 15 retrieved 12/14 tapers making up each group. This pattern has already been reported by other authors for both in vitro and retrieved micro-grooved tapers [5,14], although the taper surface of the investigated retrieved implants may be different in terms of surface topography [42], manufacturing tolerances [3], or material treatment, e.g., the coating of the taper [43]. It must be stressed that EDX analysis mainly detected the presence of titanium, aluminium, and vanadium, i.e., the main elements of the Ti-alloy. Oxygen signals, when present, were only found on the edges of the taper ridges.

Carbon peaks were also present in the spectra, which probably belonged to organic traces that had resisted the cleaning processes, as reported in Koch’s study [35]. This means that oxygen may belong to either organic compounds, titanium oxide, or both. Even assuming that all oxygen comes from titanium oxides, its limited amount should be noted. It could be hypothesised that the micromovement at the head/taper or sleeve/taper interface was so small that contact conditions were close to the partial slip regime limiting, or even avoiding, damage to the Ti-alloy passive layer and subsequent surface repassivation [18,44]. Although this study cannot demonstrate evidence for such an assumption, the current observations suggest that the micromotion amplitude within the head/taper or sleeve/taper junction may be in the order of magnitude of tens of microns [18,45,46]. Therefore, the present results suggest that affixing a sleeved ceramic head to a 12–14 micro-grooved taper of a stem left in situ does not seem to increase the risk of MACC within the sleeve/taper junction when junction stability is achieved intraoperatively. Indeed, the proper seating of the ceramic head, with or without the adapter sleeve, is a prerequisite for decreasing the risk of in vivo damage within the taper junction and, ultimately, of trunnionosis [47].

Several important limitations must be taken into account when considering the present findings: (i) the sample size of each group was 15. This limited sample size was dictated by the number of retrieved implants including sleeved ceramic heads eligible for this study found in REPO, which made up Group 3. (ii) All the analysed 12/14 tapers were micro-grooved (lightly threaded) and made of Ti-6Al-4V alloy (Ti-alloy). (iii) All the retrieved implants had short-, medium-, or long-neck heads. (iv) It was not possible to identify a perfectly matched cohort. Indeed, differences in the patients’ BMI were greater than five in three out of fifteen triads and differences in the patients’ ages were greater than 10 years in six out of fifteen triads. (v) Questionnaires assessing the patients’ activity levels were not available. (vi) The mean total implantation time was about 4 years, with the longest time being about 10 years. (vii) All the retrieved implants were cementless. (viii) Finally, the taper surface could be damaged during implant retrieval; surface roughness was not measured in 2% of the investigated regions (11 out of 540) due to the local presence of an artefact. More subtle artefacts might be generated during implant retrievals. This limitation, common to all studies investigating taper damage occurring in vivo, should have a marginal effect on the damage found on stem tapers. With these limitations in mind, the present findings do not support the hypothesis that the use of sleeved ceramic heads assembled with the used Ti-alloy micro-grooved 12/14 tapers increases the risk of MACC within the sleeve/taper junction in the short–medium term. This conclusion is only valid for short-, medium-, and long-neck head configurations implanted without the use of bone cement in patients with presumably normal activity levels. Indeed, it has been demonstrated that micromotion at the head–taper junction increases by increasing head offset and patient activity level [45,48]. In addition, the taper surface’s finishing plays a role in determining micromotion amplitude [13,49]. Therefore, it cannot be excluded that extralong-neck head configurations, different taper surface finishing (smooth or macro-grooved taper), high physical activity levels, and longer implantation time may affect junctional stability by increasing the micromotion amplitude—a key factor affecting MACC—at the sleeve/taper interface under physiological loads, with a potential detrimental effect on the progression of surface damage. Therefore, further studies should be performed to assess the impact of potentially more critical conditions on the risk of MACC occurrence within the sleeve/taper junction when a large number of retrieved implants are available.

## 5. Conclusions

The Ti-alloy/Ti-alloy sleeve/taper junction seems only mildly susceptible to severe mechanically assisted crevice corrosion.

Affixing a sleeved ceramic head, with variable neck lengths ranging up to a “long-neck”, to a Ti-alloy micro-grooved 12/14 taper of a stem left in situ does not seem to increase the risk of revision due to trunnionosis, at least in the mid-term period.

The proper seating of the sleeved ceramic head is a prerequisite for decreasing the risk of in vivo damage within the taper junction and, ultimately, of trunnionosis.

## Figures and Tables

**Table 1:** Clinical information and ceramic head configurations. Further details are available in the Supplementary Materials (Figure S2 and Table S1).

	Group 1	Group 2	Group 3	
	Clinical Information			Kruskal–Wallis
	Median (Range)			*p*-Value
Total implant. Time				
(years)	4.1 (0.2–9.6)	3.9 (0.5–10.0)	4.2 (0.4–10.2)	0.92
BMI				
(kg/m^2^)	30.1 (23.4–35.6)	29.4 (23.7–36.4)	29.6 (22.8–37.7)	0.82
Age at retrieval				
(years)	61 (35–79)	68 (30–70)	56 (38–77)	0.06
**Ceramic head configuration**				
**Number of retrieved heads**				
Diameter (mm)				
32/36/40	2003/8/4	2002/8/5	2004/8/3	/
Neck length				
S/M/L	2005/7/3	2005/6/4	2004/8/3	/

## Data Availability

Data supporting reported results are available in the Supplementary Materials.

## Supplementary Materials

The following supporting information can be downloaded at: https://www.mdpi.com/article/10.3390/ma16031067/s1, Figure S1: Workflow of the selection of retrieved hip prostheses; Figure S2: Clinical details of the 15 explant triads; Figure S3: Mean values of Goldberg scores for proximal, middle, and distal regions (error bars represent one standard deviation); Figure S4: Changes in roughness profile (taper tip on the left) in the proximal (1), middle (2), and distal regions (3). The reference profile is shown in green colour; Table S1: Reason for revision of the 45 explants; Table S2: Goldberg scores; Table S3: Percentage changes in roughness parameters.
