# Supplementary material for: Surface Analysis of Ti-Alloy Micro-Grooved 12/14 Tapers Assembled to Non-Sleeved and Sleeved Ceramic Heads: A Comparative Study of Retrieved Hip Prostheses
Source: Materials (Basel). 2023 Jan 25;16(3):1067. doi: 10.3390/ma16031067 (PMC9920329; doi:10.3390/ma16031067)
Supplement: Supplementary file 1 [file materials-16-01067-s001.zip › Table S1 Reason for revision of the 45 explants.pdf]

	Group 1	Group 2	Group 3
**Reason for first revision**		**Number of implants**	
Instability	/	8	9
Cup aseptic loosening	/	7	6
Total	/	15	15
**Reason for revision**		**Number of implants**	
Infection	7	10	9
Aseptic loosening	5	3	5
Instability	3	1	/
Perirposthetic fracture	/	1	1
Total	15	15	15
