# Supplementary figures and images for: Surface Analysis of Ti-Alloy Micro-Grooved 12/14 Tapers Assembled to Non-Sleeved and Sleeved Ceramic Heads: A Comparative Study of Retrieved Hip Prostheses
Source: Materials (Basel). 2023 Jan 25;16(3):1067. doi: 10.3390/ma16031067 (PMC9920329; doi:10.3390/ma16031067)

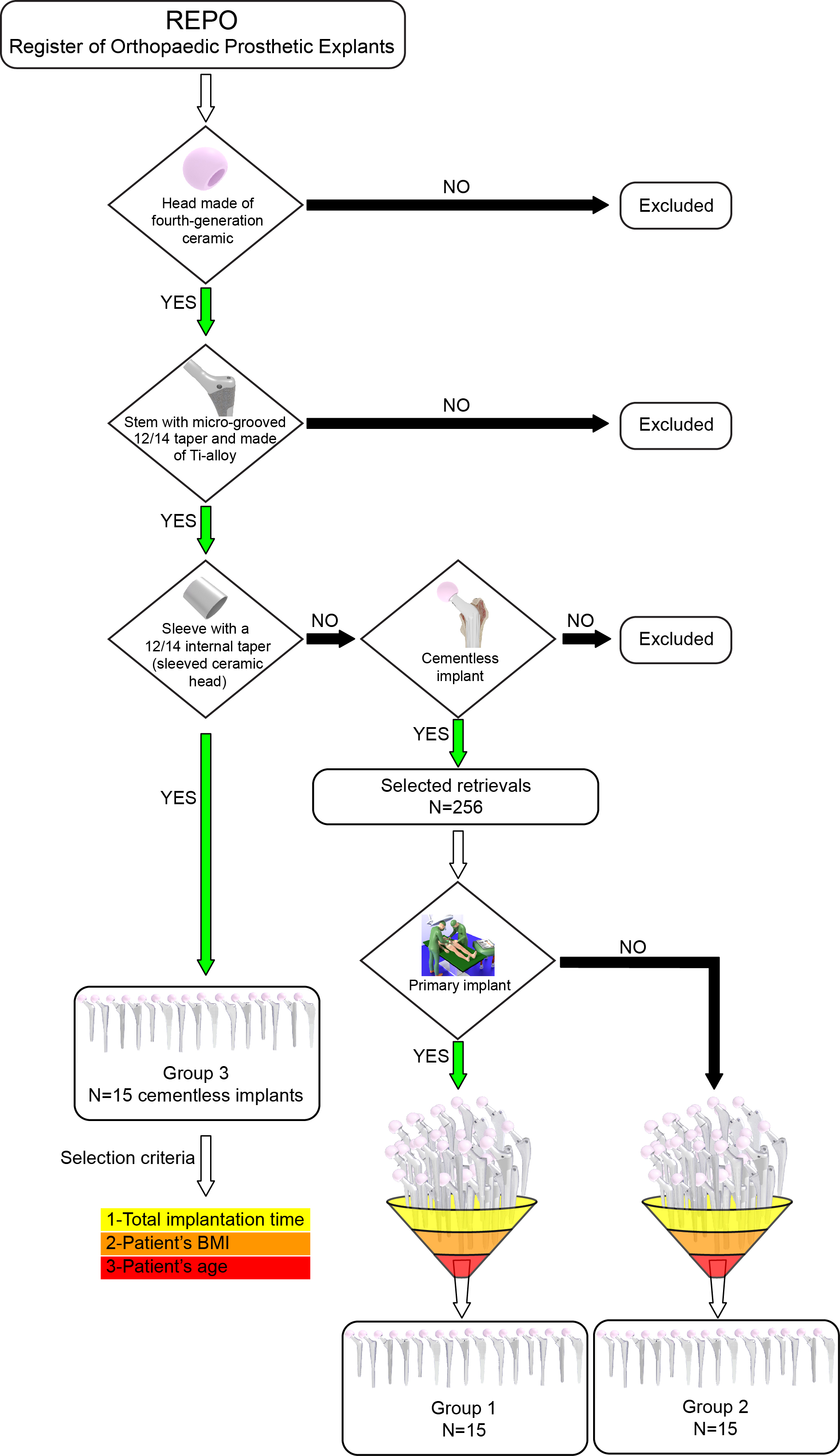

Supplement: Supplementary file 1 [file materials-16-01067-s001.zip › Figure S1 Workflow of the selection of retrieved hip prostheses.jpg]

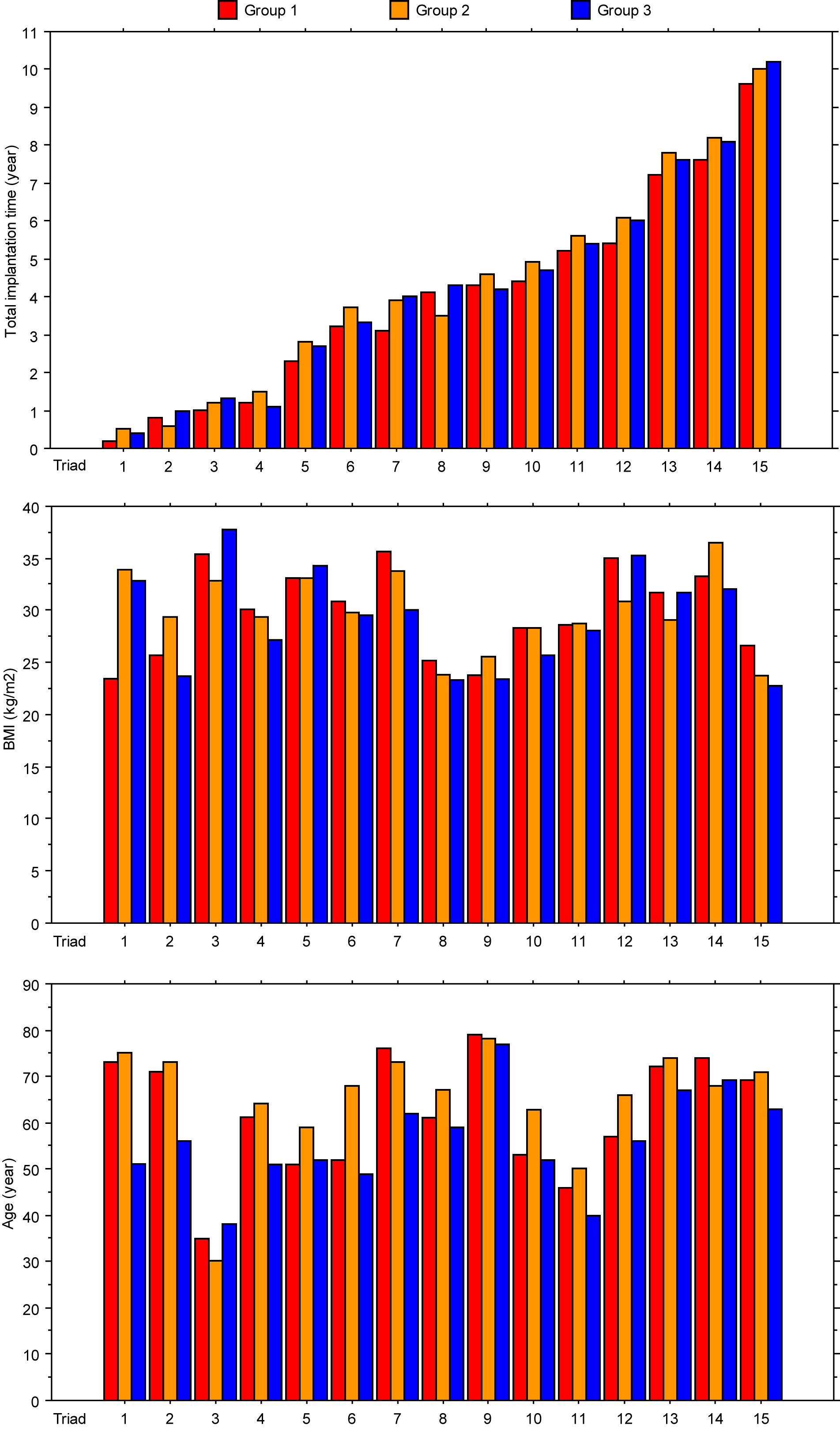

Supplement: Supplementary file 1 [file materials-16-01067-s001.zip › Figure S2 Clinical details of the 15 explant triads.jpg]

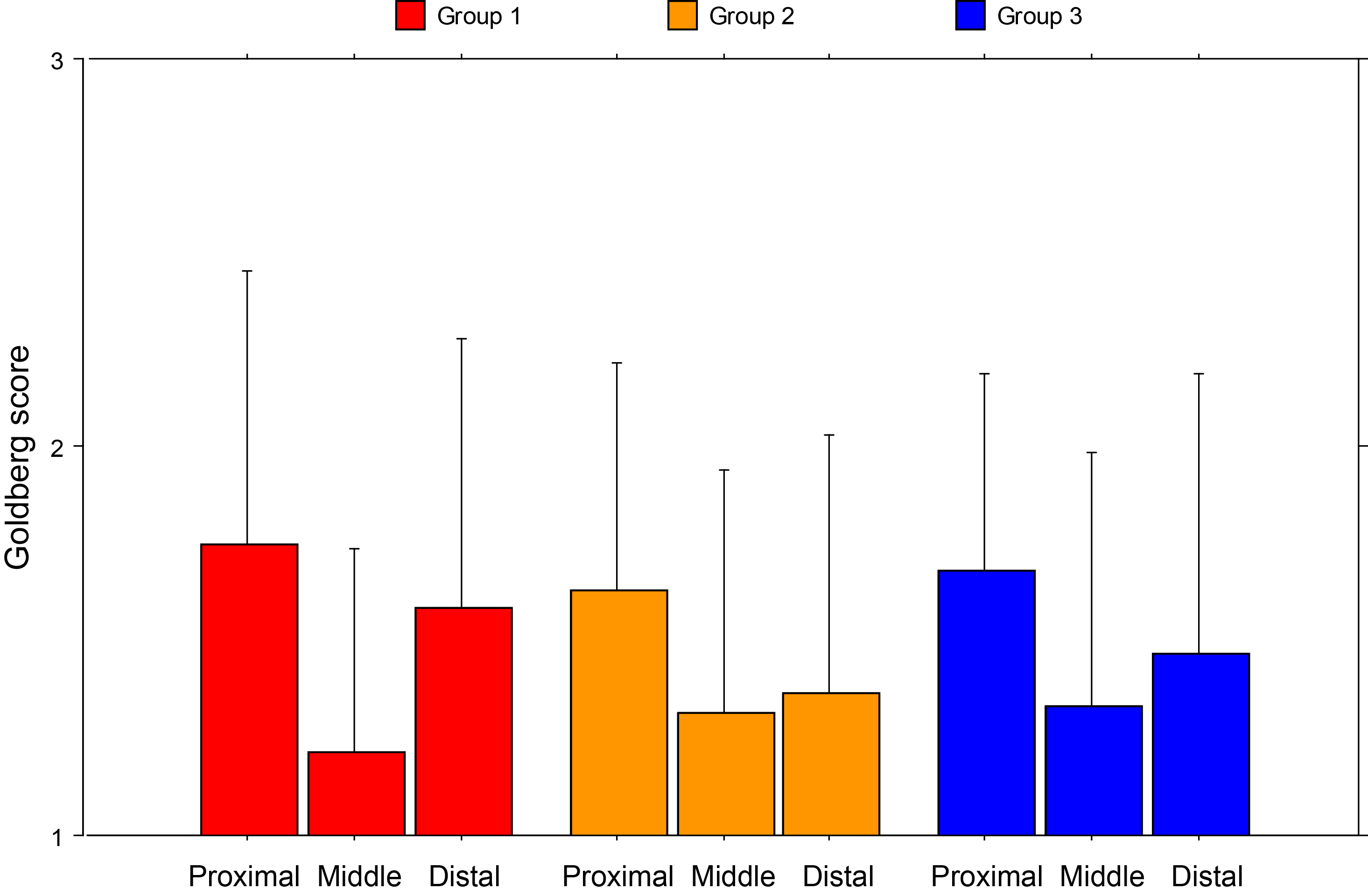

Supplement: Supplementary file 1 [file materials-16-01067-s001.zip › Figure S3 Mean values of Goldberg scores for proximal, middle, and distal regions (error bars represent one standard deviation).jpg]

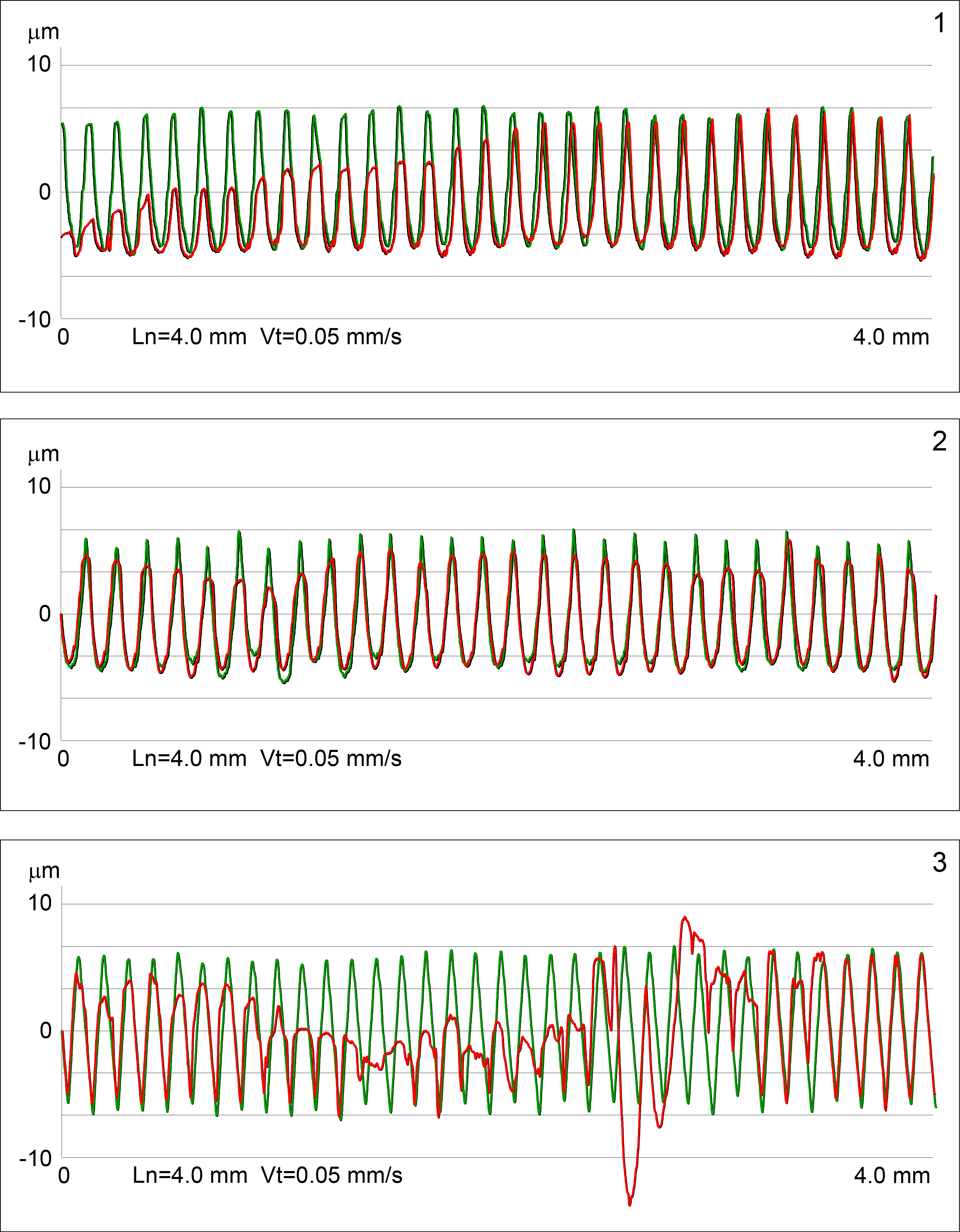

Supplement: Supplementary file 1 [file materials-16-01067-s001.zip › Figure S4 Changes in roughness profile (taper tip on the left) in the proximal (1), middle (2), and distal regions (3). The reference profile is shown in green colour.jpg]
